# A kappa opioid receptor agonist, difelikefalin, improves acute kidney injury in experimental sepsis models

**DOI:** 10.1371/journal.pone.0343693

**Published:** 2026-04-21

**Authors:** Yoshiharu Sawanobori, Daisuke Nakano, Hiroaki Kitamura, Kento Kitada, Takehiko Asaga, Norio Suzuki, Masayuki Yamamoto, Yuichi Ogino, Gotaro Shirakami, Akira Nishiyama

**Affiliations:** 1 Department of Anesthesiology, Kagawa University, Kagawa, Japan; 2 Department of Pharmacology, Kagawa University, Kagawa, Japan; 3 Division of Oxygen Biology, United Centers for Advanced Research and Translational Medicine, Tohoku University Graduate School of Medicine, Sendai, Japan; 4 Department of Integrative Genomics, Tohoku Medical Megabank Organization, Tohoku University, Sendai, Japan; 5 Department of Biochemistry and Molecular Biology, Tohoku Medical Megabank Organization, Tohoku University, Sendai, Japan; Universidade de Sao Paulo, BRAZIL

## Abstract

There is a pressing need for therapies that can lessen the severity of acute kidney injury (AKI) and subsequent critical illness. Difelikefalin, a peripherally acting kappa opioid receptor agonist, shows promise in controlling postoperative pain and chronic kidney disease-associated pruritis. Here, we report that difelikefalin attenuated AKI in experimental mouse models of critical illness. Preconditioning with difelikefalin significantly reduced oliguria in both lipopolysaccharide (LPS)-induced and cecum ligation and puncture-induced models. Moreover, difelikefalin treatment after renal ischemia/reperfusion substantially decreased the LPS-induced mortality rate on day 6 post-ischemia/reperfusion. We hypothesized that the reno-protective effects of difelikefalin are mediated through receptors expressed in either the neural system, immune cells, or the renal parenchyma. Denervation of nerves around the renal pedicle improved urine flow; difelikefalin further enhanced this improvement, regardless of renal denervation. Difelikefalin did not affect plasma cytokine levels after LPS administration at 3 and 6 hours. Renal kappa receptor expression overlapped with the pattern of neural crest-derived interstitial cells. LPS administration tended to increase cytokine expression in isolated renal kappa receptor-positive cells, and difelikefalin suppressed these changes. Overall, difelikefalin demonstrated renal protective effects against AKI in murine endotoxemia and polymicrobial sepsis models and improved survival in post-ischemia/reperfusion endotoxemia.

## Introduction

Acute kidney injury (AKI), characterized by elevated blood creatinine levels or decreased urine output, frequently occurs in clinical settings such as postoperative states and sepsis [1]. Invasive surgery itself can act as an initial trigger for AKI, causing hemodynamic instability and decreased circulating blood volume [1]. Furthermore, the risk of infection increases during the perioperative period. Sepsis resulting from postoperative infection can then become a second trigger for AKI, leading to irreversible renal dysfunction [2] and high mortality [2,3]. The underlying mechanisms of AKI are complex. For example, septic and ischemic types of AKI are presumed to have different causes [4]. Septic AKI typically involves functional changes, whereas ischemic AKI is associated with structural damage [4,5]. However, the current clinical approach to AKI treatment, regardless of cause, primarily focuses on maintaining renal blood flow with fluids and vasopressors [6]. No targeted drug therapy has been established as an effective treatment. Extensive basic research has been conducted to identify drug targets that can restore acutely injured kidneys, but this goal remains elusive [6]. Challenges in drug development include a lack of significant efficacy and unexpected safety issues in clinical trials, despite promising results in preclinical studies. One factor contributing to this translational gap is that most basic research studies administer candidate drugs before the onset of AKI, using a strategy known as preconditioning. This approach, particularly for drugs with novel targets, faces ethical and safety concerns because the study participants do not meet diagnostic criteria for AKI. Conversely, treatments initiated after the onset of AKI are often ineffective, even if they showed promise in pre-AKI treatment settings. Considering these challenges, our study aimed to examine whether any existing drug, which could potentially be used to treat patients at high risk of developing critical illness, also exhibits anti-AKI effects. Therapies using such drugs have fewer ethical and safety concerns compared with newly developed molecularly targeted drugs or repurposed drugs originally intended for other conditions.

Opioids reportedly can alleviate experimental ischemic AKI, but the specific receptors involved remain unclear [7,8]. The use of μ receptor agonists for prophylactic purposes is limited due to concerns about side effects (e.g., dependence and respiratory depression). Difelikefalin, a peripheral kappa opioid receptor agonist, is an United States Food and Drug Administration (FDA)-approved antipruritic drug for patients with chronic kidney disease; it also is under investigation for use as a postoperative analgesic [9]. Furthermore, difelikefalin may counteract the development of septic AKI through its diuretic effect, its ability to suppress excessive inflammatory cytokine release, and its analgesia-related capacity to attenuate sympathetic hyperactivity [10]. We hypothesized that difelikefalin, if administered in situations causing AKI/critical illness—specifically, after the initial insult (e.g., surgery and potential renal ischemia/reperfusion) and before a secondary insult, such as infection—could prevent or attenuate AKI. Therefore, we examined whether difelikefalin could prevent septic AKI, including postoperative endotoxemia, and explored its potential mechanism in mouse models of experimental sepsis.

## Materials and methods

### Animals

All experimental procedures adhered to the guidelines for animal care and use established by Kagawa University. Animals were housed in an environment with a temperature of 24 ± 1°C, humidity of 55% ± 5%, and a 12-hour light-dark cycle. They had free access to water and standard chow (MF; Oriental Yeast Co., Ltd., Japan) except during general anesthesia. *Sprague–Dawley* rats or C57Bl/6J male mice were obtained from Clea Japan, Inc. (Tokyo, Japan) and acclimated in our animal facility for at least 1 week until they reached 7–10 weeks of age, weighing 250–350 g or 20–25 g, respectively. Animals were kept warm using a heating pad during general anesthesia lasting longer than 1 hour. The euthanasia was conducted based on pre-defined criteria, by using either carbon dioxide, cervical dislocation or bloodletting under isoflurane anesthesia for mice experiments, or pentobarbital injection for rat experiment using inaction anesthesia.

### Chemicals

Difelikefalin was generously provided by Maruishi Pharmaceutical Co., Ltd. (Osaka, Japan) as a 10 mg/ml aqueous saline solution. Lucifer Yellow (LY) was purchased from Invitrogen (Waltham, MA, USA). Lipopolysaccharide (LPS O-55. B-5) and thiobutabarbital (Inactin) were obtained from Sigma-Aldrich (St. Louis, MO, USA). Isoflurane was acquired from Abbott Japan (Tokyo, Japan).

### Assessments of difelikefalin effects on blood pressure, pulse rate, and urine flow rate in normal rats

In this experiment only, rats were used instead of mice because it was technically challenging to measure blood pressure from cannulated arteries and hourly urine output stably for extended periods under general anesthesia in mice. Cannulation of the trachea, internal jugular vein, femoral artery, and bladder was performed under inactin anesthesia. Tracheal cannulation ensured airway patency, and rats were managed with spontaneous ventilation. Infusion fluids and drugs were injected using a 20-cc syringe (Terumo, Tokyo, Japan) attached to a high-precision micro syringe pump (CXF101, TGK, Tokyo, Japan) through a catheter inserted into the internal jugular vein. Mean blood pressure and pulse rate were measured using LabChart software (ADInstruments, Sydney, Australia) and a PowerLab 8/35 data acquisition device (ADInstruments) connected to a pressure transducer. After catheterization, a saline infusion of 3 ml/kg/h was initiated; the rats were allowed to recover for 1 hour, designated as hour 0. At the end of hour 0, the saline infusion was reduced to a maintenance dose of 2 ml/kg/h, establishing the 1-hour baseline. Blood pressure, pulse rate, and urine output were measured hourly thereafter until hour 7. Saline or difelikefalin (1 mg/kg/2h) was administered, from hour 1 to hour 3.

### In vivo imaging to quantify urine output after experimental sepsis

Five hours after experimental sepsis induction, mice were anesthetized with pentobarbital and isoflurane, then subjected to tracheal and internal jugular vein cannulation. A small flank incision was made to expose one kidney, which was then adhered to a coverslip using glue. Mice were allowed to recover for 30 minutes; in vivo imaging was performed 6 hours after experimental sepsis induction. The imaging procedure was carried out as previously described [11]. Briefly, multiphoton excitation was achieved with a femtosecond laser (Chameleon Ultra-II MP laser, Coherent Inc., Santa Clara, CA), and 512 x 512 pixel images were acquired with an upright multiphoton laser scanning microscope (FV-1000MPE, Olympus, Tokyo, Japan) equipped with a 25x water immersion objective lens (numerical aperture 1.05). Irradiation with 720 nm and 860 nm lasers was utilized to obtain z-stack images at 1.5-µm intervals from the kidney surface to a depth of 50 µm, thereby allowing visualization of proximal and distal nephron arrangement. LY freely passes through the glomerulus, is minimally reabsorbed in the renal tubules (similar to inulin), and emits sharp fluorescence at 430–450 nm. We observed the LY signal by using 860 nm laser irradiation to capture time-lapse images every 2 s for 300 s after intravenous administration of LY. By identifying distal nephrons in z-stack images and measuring the time from LY injection to the appearance of fluorescence within the distal nephron on time-lapse images, we determined urine flow velocity in the renal tubules.

We performed three experiments using this in vivo imaging technique to measure urine flow velocity. First, we investigated the pre-treatment effect of difelikefalin on the experimental sepsis-induced decrease in urine flow velocity. Difelikefalin (0.3 or 1.0 mg/kg in 10 ml/kg saline) or saline (10 ml/kg) for the control group was intravenously injected via the retroorbital vein under brief isoflurane anesthesia, 1 hour before experimental sepsis induction with LPS or CLP. Experimental sepsis was induced by injecting LPS or performing cecum ligation and puncture (CLP) in mice. LPS (5 mg/kg) was intraperitoneally injected [11]. CLP and sham operations were performed under isoflurane anesthesia [12]. After a cecal ligation, two puncture was made by using a 21-gauge needle and the ligated cecum was gently squeezed to express a 1-mm column of fecal material. One mL of saline was injected intraperitoneally before closing surgical window. Next, to observe the long-term effect of difelikefalin, we intravenously injected difelikefalin (1.0 mg/kg in 10 ml/kg saline) or saline (10 ml/kg) for the control group via the retroorbital vein under brief isoflurane anesthesia, 3 or 7 days before experimental sepsis induction with LPS. Finally, we investigated the pretreatment effect of difelikefalin on the experimental sepsis-induced decrease in urine flow velocity after bilateral denervation. Bilateral renal denervation was performed 7 days before the onset of experimental sepsis. After the induction of isoflurane anesthesia, mice underwent a small bilateral flank incision to expose the renal hilum. The renal nerves were identified using a stereomicroscope and cut with sharp forceps, and a small amount of 10% phenol was applied on a cotton swab [13].

To confirm successful renal denervation, we collected the kidneys after in vivo imaging and measured the decrease in tissue norepinephrine concentration.

### Quantification of urine output in awake mice after LPS-induced experimental sepsis

Urine sample collection was performed using a separate group of mice (i.e., not used for other experiments) to eliminate the potential impact of a different environment (metabolic cages) on other parameters. Mice were acclimated in metabolic cages for 24 hours prior to urine collection. Under brief isoflurane anesthesia, mice in the normal control and LPS vehicle groups received saline, whereas mice in the CR0.3 LPS and CR1.0 LPS groups received 0.3 or 1 mg/kg of difelikefalin, respectively, through the retroorbital vein. One hour after saline or difelikefalin injection, we intraperitoneally injected 5 mg/kg of LPS. Using glass vials, we collected urine from the mice for the next 6 hours. Urine volume was measured by weight (1 g/mL).

### Survival assessment after the onset of renal ischemia and LPS-induced experimental sepsis

We developed an experimental mouse model to mimic sepsis after major invasive surgery, a common clinical scenario, then observed the effect of difelikefalin administration on survival. After unilateral nephrectomy, the remaining kidney was subjected to 30 minutes of ischemia followed by reperfusion under isoflurane anesthesia. On days 1, 3, and 5, saline or difelikefalin (0.3 mg/kg) was administered; LPS (15 mg/kg) was administered on day 6. The survival rate was monitored by the authors every 12 hours until 144 hours, and every 24 hours thereafter until day 952, the day the last mouse reached the criteria noted below. Humane endpoints were used to minimize suffering. Mice were observed at every survival rate monitoring, and if all of the following criteria were observed, they were immediately euthanized by carbon dioxide: unable to move or move only when provoked, significant labored breathing, ruffled fur, and weight loss of 30% or more. Euthanasia was included in the survival rate analysis (13/16 mice). The three mice found dead between each observation period within 36 hours because of severe endotoxemia after renal ischemia/reperfusion.

### Plasma cytokine measurement

Plasma sample collection was performed using a separate group of mice (i.e., not used for other experiments) because any surgical procedure can affect plasma cytokine levels. Blood samples were collected from the retroorbital vein at 3 and 6 hours after LPS administration. Plasma levels of tumor necrosis factor (TNF)-α, interleukin (IL)-6, IL-10, and interferon (IFN)-γ were measured using Quantikine enzyme-linked immunosorbent assay kits (R&D Systems, Minneapolis, MN, USA).

### Immunofluorescence

For this experiment, *P0-Cre tdTomato* mice expressing tdTomato in most renal interstitial cells were taken from the colony maintained in our laboratory. Kidneys were cryosectioned at 10 µm, captured on slides, then fixed in formalin for 1 hour and blocked with 1% bovine serum albumin in phosphate-buffered saline for 1 hour. Samples were incubated overnight with primary antibody: anti-KOR1 (D-8, sc-374479, Santa Cruz, Dallas, TX, USA). Samples were then washed using 1% bovine serum albumin in phosphate-buffered saline and incubated for 3 hours with secondary antibody: Alexa488-conjugated anti-mouse IgG (1:400; Invitrogen). Data were analyzed by two-photon microscopy (FV1000-MPE, Olympus).

### Isolation of KOR1-positive cells from the kidney

Untreated C57Bl/6J mice were euthanized by bloodletting under isoflurane anesthesia, then transcardially perfused with cold phosphate-buffered saline. Both kidneys were isolated and minced in an ice-chilled dish. The minced kidneys were then digested with an enzyme cocktail containing Liberase (0.52 U/mL), DNase 1 (3 U/mL), and hyaluronidase (10 μg/mL) in Hank’s Balanced Salt Solution (2 mL/kidney) at 37°C for 60 minutes [14]. The lysate was then washed and passed through a 35-μm cell strainer; KOR+ cells were isolated from the resulting mixture using a magnetic-activated cell sorting system (Miltenyi Biotech, Bergisch Gladbach, Germany) with an anti-KOR1 antibody (Santa Cruz) and magnetic beads conjugated to a secondary antibody.

### RT-PCR

One-third of each kidney was sectioned at the lower pole and immersed overnight in RNAlater (Sigma-Aldrich). These samples were then used for reverse transcription polymerase chain reaction (RT-PCR) analysis. We analyzed the levels of 18S rRNA and mRNA of TNF-α, chemokine (C-C motif) ligand 3 (CCL3), and IL-10 using a 7300 Fast Real-Time PCR System (Applied Biosystems, Foster City, CA, USA). The mouse primer sequences (forward and reverse) were: 18S, 5′-GTAACCCGTTGAACCCCATT-3′ and 5′-CATCCAATCGGTAGTAGCG-3′; TNFα, 5ʹ-TGGCACCACTAGTTGGTTGTCT-3ʹ and 5ʹ-AGCCTGTAGCCCACGTCGTA-3ʹ; CCL3, 5ʹ-TGCCCTTGCTGTTCTTCTCT-3ʹ and 5ʹ-GATGAATTGGCGTGGAATCT-3ʹ; and IL-10, 5ʹ-GGCGCTGTCATCGATTTCTC-3ʹ and 5ʹ-TGGCCTTGTAGACACCTTGGT-3ʹ. Relative mRNA expression levels were determined using the 2^−ΔΔCt^ method. ddCt values were calculated using data from control mice.

### Statistical analysis

We did not perform an a priori power analysis for this study. Instead, we used sample sizes that previous studies had shown were sufficient to detect biologically meaningful differences. Results are expressed as means ± standard deviation. Statistical significance was assessed using Student’s *t*-test for 2 groups comparison and one-way analysis of variance followed by Tukey’s multiple comparison test for multiple groups comparison. All statistical analyses were performed using GraphPad Prism 6 (GraphPad Software Inc., La Jolla, CA, USA). P-values <0.05 were considered statistically significant.

## Results

### Effects of difelikefalin on blood pressure, pulse rate, and urine flow rate in normal rats

Continuous administration of difelikefalin (1 mg/kg for 2 hours) rapidly increased the hourly urine volume in anesthetized rats. This diuretic effect gradually subsided after the infusion ended and became statistically insignificant at 3 hours post-administration (S1 Fig). These findings confirmed that the drug did not induce an anti-diuretic effect with the potential to exacerbate AKI; instead, it had a diuretic effect. Continuous difelikefalin administration did not alter blood pressure or pulse rate relative to baseline measurements.

### Effects of difelikefalin on urine flow after experimental sepsis induction

**Fig 1:**
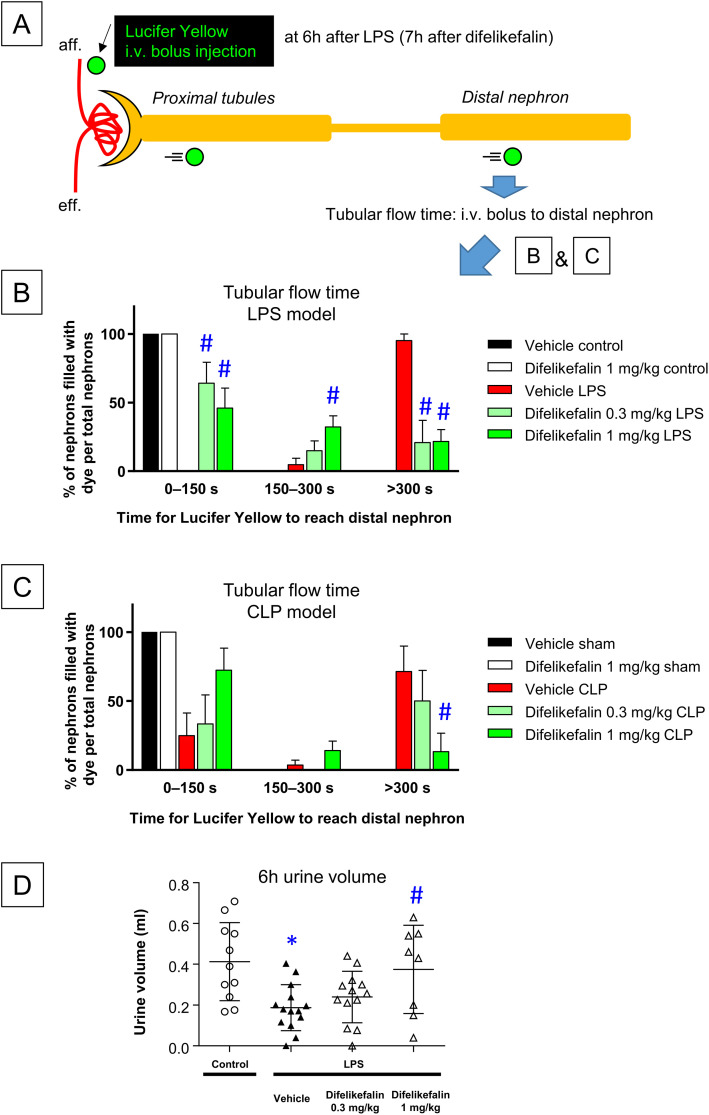
Difelikefalin protects against septic AKI. A: Rationale for analyzing tubular flow rate by in vivo imaging. Intravenously (i.v.) injected fluorescent dye, Lucifer Yellow, flows into afferent arterioles, is freely filtered from glomeruli, passes through the proximal tubular lumen, and reaches the lumen of distal nephrons. The interval from i.v. injection to distal lumen arrival was measured and is described as tubular flow time in Fig 1B and 1C. B: Tubular flow time at 6 hours after LPS injection with and without difelikefalin (0.3 or 1 mg/kg)(n = 6-8). C: Tubular flow time at 6 hours after CLP with and without difelikefalin (0.3 or 1 mg/kg)(n = 6-8). D: Urine volume for 6 hours after LPS injection with and without difelikefalin (0.3 or 1 mg/kg)(n = 8-14). aff, afferent arterioles; eff, efferent arterioles; LPS, lipopolysaccharide; CLP, cecum ligation and puncture. *p < 0.05 vs. control, #p < 0.05 vs. vehicle LPS or CLP.

We investigated the renal effects of experimental sepsis using a real-time intravital imaging system with two-photon laser microscopy. LY, a small molecule freely filtered from the glomeruli, was used in combination with two-photon microscopy to visualize tubular flow. We measured the time required for LY to reach the distal nephron, as illustrated in Fig 1A; a shorter time indicates a faster tubular flow rate. As noted in the Methods section, all mice were pre-treated with saline 1 hour before surgery. In mice treated with saline alone, LY reached the distal nephron within 150 s for all nephrons in the imaging window (Fig 1B). Similar results were observed in mice treated with difelikefalin alone. Difelikefalin administration 1 hour before LPS (5 mg/kg) ameliorated the LPS-induced decrease in tubular flow rate. Six hours after LPS administration, the percentage of distal nephrons reached by LY within 150 s was 0% in the control group; this percentage increased to 64% and 46% with 0.3 mg/kg and 1 mg/kg of difelikefalin, respectively. The percentage of distal nephrons not reached by LY even after 300 s decreased from 95% to 21% and 22%, respectively. Six hours after CLP-mediated induction of sepsis, LY reached only 25% of the total distal nephrons within 150 s in the control group; it did not reach 71% of the distal nephrons even after 300 s (Fig 1C). In contrast, whereas 1-hour pre-administration of difelikefalin (0.3 mg/kg) before CLP did not improve the tubular flow rate, pre-administration of difelikefalin (1 mg/kg) increased the percentage of distal nephrons reached within 150 s and decreased the percentage of distal nephrons not reached by LY even after 300 s. At six hours after sham surgery, all distal nephrons were reached by LY within 150 s in both the control group and the difelikefalin (1 mg/kg) pre-treatment group.

We assessed awake mice to confirm the effects observed during in vivo imaging. LPS administration reduced the 6-hour urine volume in these mice. Difelikefalin at 1 mg/kg dosage significantly attenuated this LPS-induced decrease in urine volume, thereby reproducing the data from anesthetized mice (Fig 1D).

### Preconditioning effect of difelikefalin on tubular flow rate

**Fig 2:**
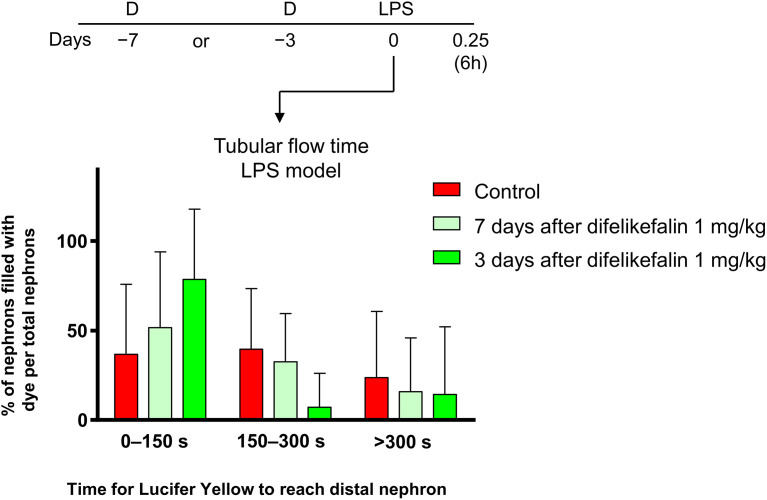
Tubular flow time in mice treated with difelikefalin either 3 or 7 days prior to LPS injection (n = 6-8). Difelikefalin was administered 3 and 7 days before LPS-induced experimental sepsis, respectively, and tubular flow time was observed 6 hours after LPS administration using in vivo imaging. The “Control” group received no difelikefalin as a preconditioning treatment. LPS, lipopolysaccharide.

We measured the duration of difelikefalin-mediated oliguria inhibition. There was a tendency on difelikefalin-mediated effects on suppressing the decrease in tubular flow rate when administered 3 and 7 days before LPS administration; however, neither preconditioning timing achieve statistically significant difference (Fig 2).

### Effects of difelikefalin on mortality on a post-surgical endotoxemia model

**Fig 3:**
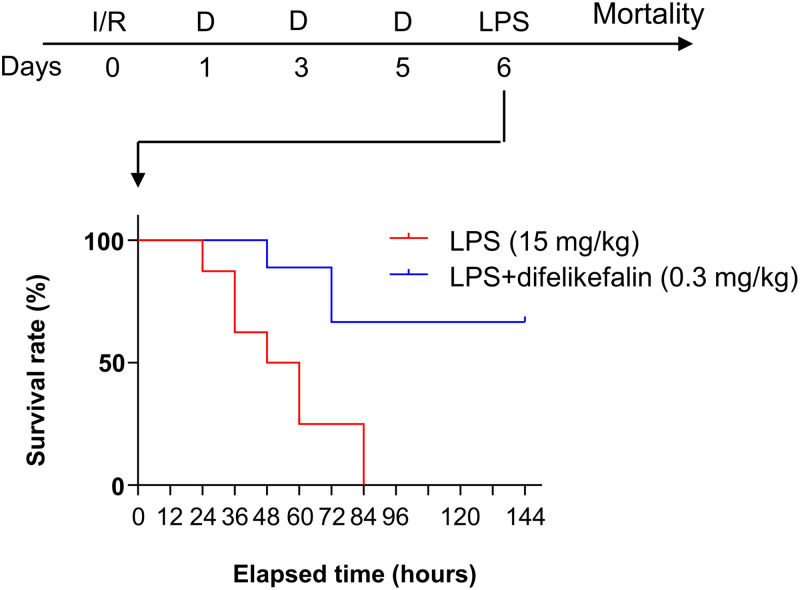
Effects of difelikefalin (0.3 mg/kg at day 1, 3 and 5 days after ischemia/reperfusion) on survival rate in mice experiencing renal ischemia/reperfusion 6 days before LPS injection (n = 8). I/R: renal ischemia and reperfusion surgery; D: difelikefalin 0.3 mg/kg i.v. administration; LPS: lipopolysaccharide 15 mg/kg i.p. administration.

After the onset of renal ischemia and LPS-induced experimental sepsis, the median survival time in the control group was 54 hours. More than 80% of the mice in the difelikefalin treatment group survived this acute phase (Fig 3). Five of the eight difelikefalin-treated mice surviving beyond 144 hours showed no visible abnormalities and lived for more than 2 years, suggesting that their lifespan was normal.

### Mechanism of difelikefalin-induced reno-protection

**Fig 4:**
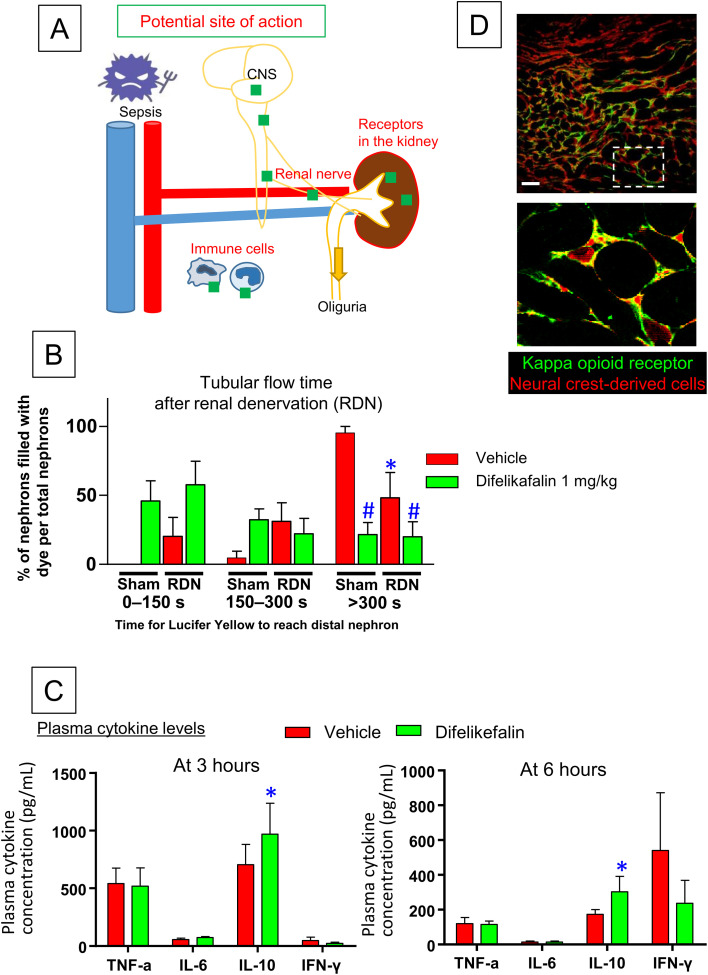
Potential sites of action for difelikefalin-induced reno-protection. A: A cartoon illustrating potential sites of kappa receptor expression (green square). B: Tubular flow time in mice that underwent renal denervation or sham operation, at 6 hours after LPS (n = 6-8). C: Plasma levels of TNF-α, IL-6, IL-10, and IFN-γ at 3 and 6 hours after LPS administration (n = 7-10). D: Immunofluorescence of kappa opioid receptors (green) in the mouse kidney. Neural crest-derived renal interstitial cells expressing tdTomato are shown in red. Scale bar: 50 μm. *p < 0.05 vs. sham vehicle, #p < 0.05 vs. vehicle in procedure. CNS: central nervous system; RDN: renal denervation.

Next, we examined the site of action for the reno-protective effects of difelikefalin. We hypothesized three potential pathways: 1) neural KOR1-dependent, 2) immune KOR1-dependent, and 3) renal KOR1-dependent (Fig 4A). In peripheral tissues, KOR1 is expressed in afferent sensory nerves that extend to the dorsal root ganglia [15], which originate from neural crest cells.

To investigate the contribution of the neural KOR1-dependent pathway to difelikefalin-induced reno-protection, we performed renal denervation, which reportedly can reduce kidney disease-associated pain [16,17]. Denervation was confirmed by a substantial decrease in renal noradrenaline content (S2 Fig). Thus, renal denervation presumably inhibited both afferent sensory signals from the kidney and efferent sympathetic nervous regulation of the kidney. Multiphoton measurement of tubular flow in LPS-injected mice revealed that renal denervation itself ameliorated the LPS-induced decrease in tubular flow rate (Fig 4B). Difelikefalin injection improved urine flow rate in both non-denervated and denervated animals, suggesting that the reno-protective effect of difelikefalin is not dependent on afferent nervous output from the kidney or efferent nervous input to the kidney.

We examined the effects of difelikefalin on cytokine release from immune cells. Plasma cytokine levels dramatically changed after LPS injection (Fig 4C). The results showed no significant differences in the levels of TNF-α, IL-6, and IFN-γ. However, difelikefalin significantly increased the level of IL-10, an anti-inflammatory cytokine, at both 3 and 6 hours after LPS injection.

Subsequent evaluation of renal KOR1 expression revealed positive immunoreactivity in interstitial cells. The KOR1 signal partially overlapped with the tdTomato-derived signal in *P0-Cre:R26t* mice, which express tdTomato in neural crest-derived cells that constitute most of the renal interstitial cell population (Fig 4D) [18].

**Fig 5:**
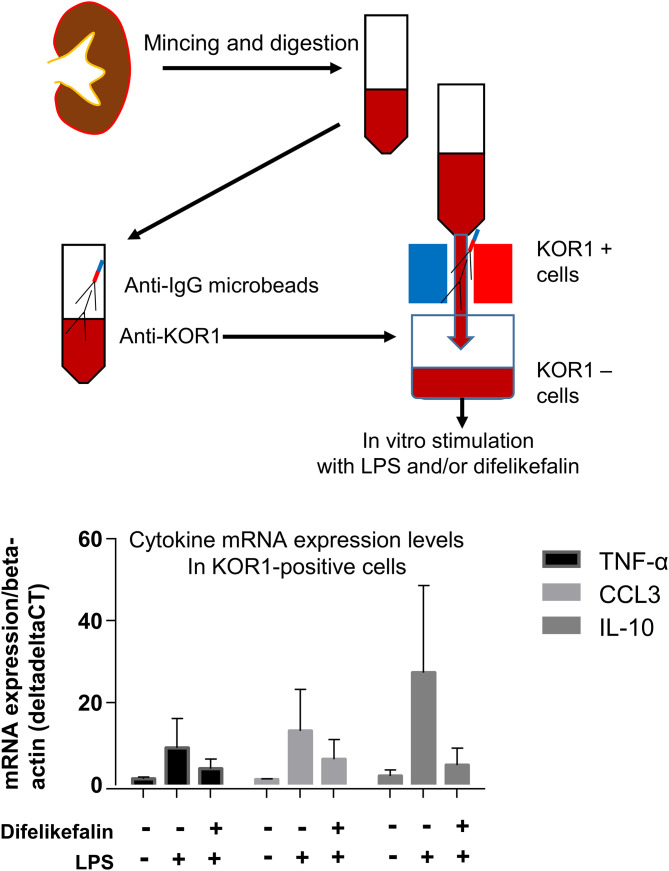
Cytokine expression levels in cells isolated from mouse kidneys and then incubated with LPS and difelikefalin ex vivo (n = 11-12 for TNF-α and CCL3, and n = 3-4 for IL-10). Top cartoon depicts the isolation of kappa opioid receptor-expressing cells. *p < 0.05 vs. no treatment, #p < 0.05 vs. LPS without difelikefalin. KOR: kappa opioid receptor; mRNA: messenger ribonucleic acid; TNF-α: tumor necrosis factor-α; CCL3: chemokine CC motif ligand 3; IL-10: interleukin 10; LPS: lipopolysaccharide.

Finally, we isolated KOR1-positive cells from the kidneys of untreated control mice and incubated them with LPS, in the presence and absence of difelikefalin (Fig 5). Several cases showed strong responses to LPS, while difelikefalin showed no such cases. Noted that no statistically significant differences were detected in the experiments using these isolated kappa-positive cells.

All raw data is available in the S1 Table.

## Discussion

Sepsis and AKI often occur in the intensive care unit among patients who have undergone surgery, and both are common causes of hospital mortality. Many efforts to combat septic AKI in hospitals have successfully reduced the mortality rate; however, it remains unacceptably high [3]. One potential management approach involves the use of prophylactic treatments, known as preconditioning. Several candidates, such as LPS, could be used for this purpose [4]. A limitation of this approach is the ethical concern surrounding prophylactic interventions, especially when they include drug treatment or endotoxin. Therefore, we aimed to identify a candidate drug for anti-septic AKI treatment that could be frequently used in patients with a high risk of septic AKI. Difelikefalin, a kappa opioid receptor agonist, may prevent sepsis-induced critical illness due to its expected diuretic [19] and anti-inflammatory [20] effects. In this study, we found that a kappa opioid receptor agonist, recently FDA-approved for anti-pruritus purposes, exerted prophylactic effects against septic AKI in mouse models. Notably, the drug improved the survival rate of animals subjected to sepsis induction after an episode of ischemia/reperfusion. Because kappa opioid receptor agonists can reasonably be used in patients with renal disease who have a high risk of sepsis, the protective effect demonstrated in this study provides insights regarding a potential therapeutic strategy for preventing septic AKI.

The mechanism by which kappa opioid receptor agonists protect the kidney against AKI appears to be multifaceted and complex. In the present study, difelikefalin exerted a strong diuretic effect and mild anti-inflammatory effects during endotoxemia; these effects gradually faded within 7 days. The kappa opioid receptor is expressed in the dorsal root ganglia [21], nerves responsible for sensory function, and the hypothalamus [22]. Its well-known physiological functions comprise regulating analgesia, sedation, and pruritus through receptors expressed by neurons in the dorsal root ganglia. Despite the long history of kappa opioid receptor research, it remains unclear whether the activation of opioid receptors in these neurons can protect the kidney against septic AKI. This uncertainty has arisen because the use of mu opioid receptor agonists (e.g., morphine and fentanyl) as analgesics requires special care in patients with reduced renal function [23,24].

Kappa receptor stimulation on sensory nerves may trigger afferent-to-efferent nervous stimulation. However, the protective effects of difelikefalin persisted even with renal denervation, suggesting that its impact on efferent renal autonomic (sympathetic) nervous regulation was limited. Another possible mechanism for the reno-protective effect of difelikefalin is that the compound activates kappa receptors expressed on non-neuronal cells, such as immune cells and renal parenchymal cells. Notably, difelikefalin shifted plasma cytokine levels towards a milder inflammatory pattern. This shift might reflect modulation of sensory nerve signals to the central nervous system or hypothalamic neurons, potentially driving an anti-inflammatory response through afferent (non-renal) nervous regulation [25,26], or direct effects of difelikefalin on the kappa opioid receptors expressed on immune cells [26,27]. In any case, the effects on the cytokine storm were only partial and appear insufficient to fully explain the protective effects of difelikefalin against AKI. We also detected kappa opioid receptors in renal medullary interstitial cells, and stimulation of these receptors in isolated renal interstitial cells showed a trend to attenuate cytokine expression. Hato et al. [28] reported that LPS exposure suppresses de novo transcription/translation (with the exception of inflammatory cytokines) in renal tissue, thereby accelerating AKI. Difelikefalin suppressed the occurrence of LPS-induced increase in cytokine expression. Thus, difelikefalin may induce quiescence of highly transcribed/translated cytokines during endotoxemia. These effects may also contribute to its renoprotective effects in mouse models of AKI.

Difelikefalin significantly improved the survival rate of LPS-treated mice at 1 week after renal ischemia/reperfusion injury. Because we induced renal ischemia in normal young mice, we presume that endotoxemia caused AKI, leading to death. Difelikefalin administration may have attenuated septic AKI, as demonstrated in our in vivo imaging and metabolic cage experiments, and protected the mice from a lethal event. It is also possible that difelikefalin accelerated kidney recovery after ischemia/reperfusion injury, and that the subsequent endotoxemia caused milder AKI. This presents an intriguing approach for preventing AKI progression to acute kidney disease and chronic kidney disease. Future studies should investigate whether difelikefalin influences renal recovery after ischemia/reperfusion injury, potentially by supporting renal epithelial proliferation and inhibiting renal inflammation.

In conclusion, we have demonstrated that the kappa opioid receptor agonist difelikefalin exerts protective effects against septic AKI in mice. This drug, which can be prescribed for other purposes, may offer protection against septic AKI when used in a prophylactic manner.

## Supporting information

S1 Fig A diuretic effect of difelikefalin in anesthetized rats. Intravenous infusion of difelikefalin (0.5 mg/kg/h) induced a significant increase in urine output collected from a bladder catheter in inactin-anesthetized rats. Values are presented as means ± SEMs. *p < 0.05 compared with the control group; two-way ANOVA followed by Sidak’s multiple comparison tests.
(TIF)

S2 Fig Renal noradrenaline level with and without renal denervation. RDN: renal denervation. #p < 0.05 vs. sham.
(TIF)

S1 Table All raw data used to create the figures. (XLSX)
