# Supplementary figures and images for: A kappa opioid receptor agonist, difelikefalin, improves acute kidney injury in experimental sepsis models
Source: PLoS One. 2026 Apr 21;21(4):e0343693. doi: 10.1371/journal.pone.0343693 (PMC13098930; doi:10.1371/journal.pone.0343693)

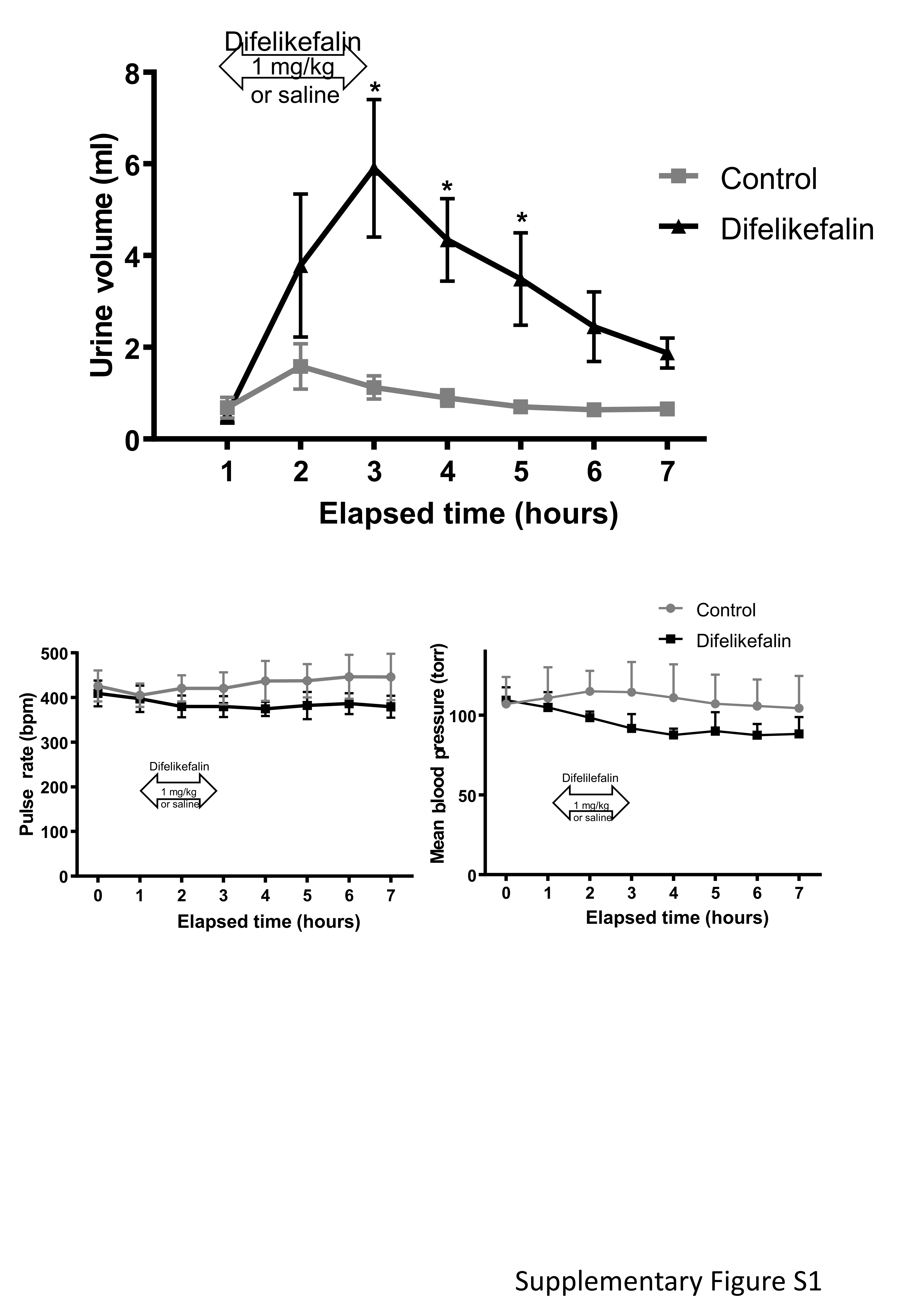

Supplement: S1 Fig — Intravenous infusion of difelikefalin (0.5 mg/kg/h) induced a significant increase in urine output collected from a bladder catheter in inactin-anesthetized rats. Values are presented as means ± SEMs. *p < 0.05 compared with the control group; two-way ANOVA followed by Sidak’s multiple comparison tests. (TIF) [file pone.0343693.s001.tif]

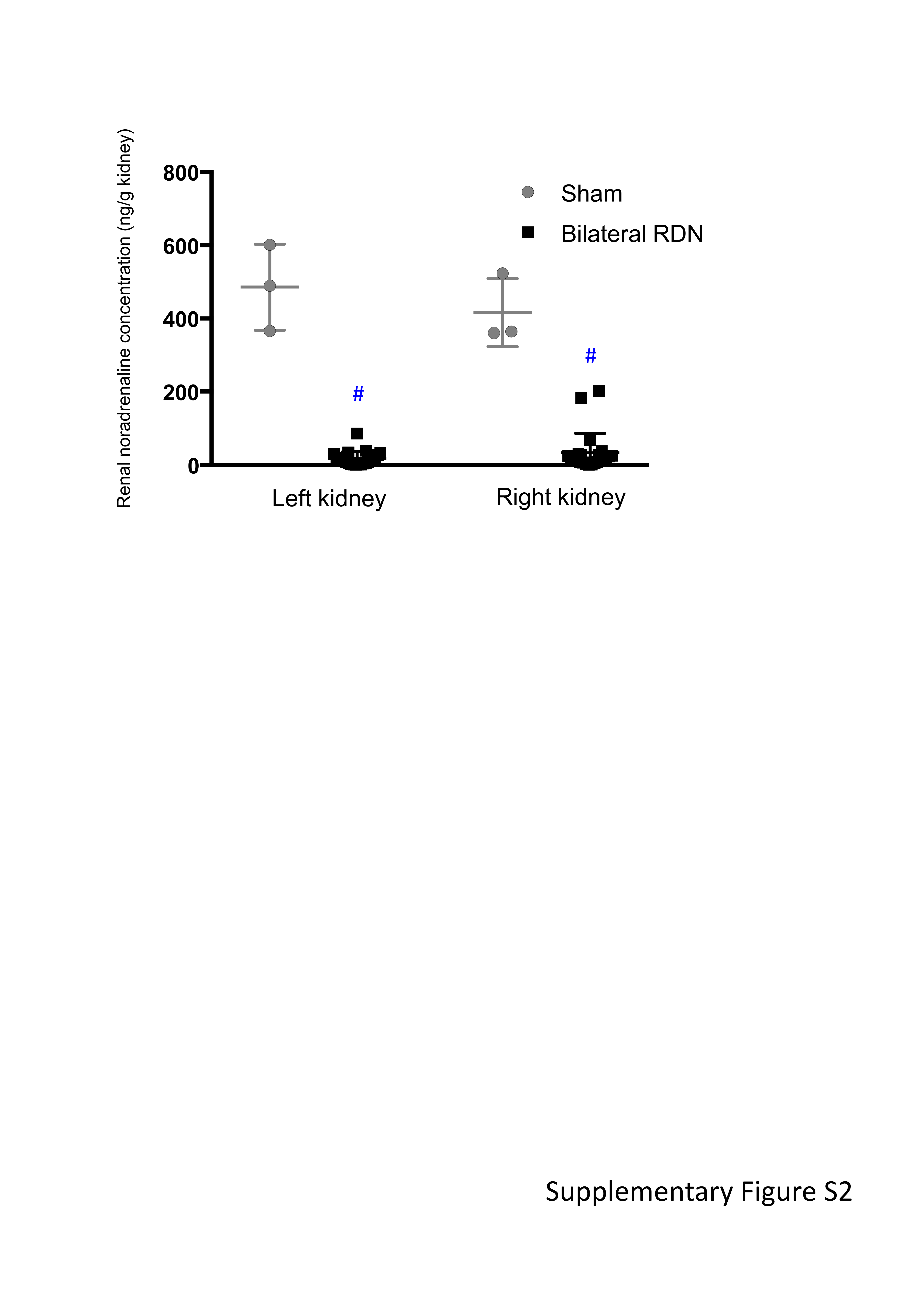

Supplement: S2 Fig — RDN: renal denervation. #p < 0.05 vs. sham. (TIF) [file pone.0343693.s002.tif]
